# MiR-125b regulates endometrial receptivity by targeting MMP26 in women undergoing IVF-ET with elevated progesterone on HCG priming day

**DOI:** 10.1038/srep25302

**Published:** 2016-05-04

**Authors:** Cheng Chen, Yue Zhao, Yang Yu, Rong Li, Jie Qiao

**Affiliations:** 1 Reproductive Medical Centre, Department of Obstetrics and Gynecology, Peking University Third Hospital, Beijing, 100191, China; 2 Key Laboratory of Assisted Reproduction, Ministry of Education, Beijing, 100191, China; 3 Beijing Key Laboratory of Reproductive Endocrinology and Assisted Reproductive Technology, Beijing, 100191, China

## Abstract

On the women undergoing IVF-ET with elevated progesterone on human chorionic gonadotrophin priming, the assisted reproductive technology outcome is poor. But, due to the unknown mechanism of this process, no effective method has been found to overcome this difficulty. Here, we investigated the roles of miR-125b and its target gene, MMP26, in endometrial receptivity (ER) in these women. The expression of miR-125b was significantly up-regulated in EECs in women with elevated progesterone during the window of implantation, and it showed a progesterone-dependent effect *in vitro*. Similarly, the expression of miR-125b was significantly up-regulated in the preimplantation period, and was down-regulated in the implantation period and the post-implantation period in mouse EECs. In addition, miR-125b showed a greater decrease at implantation sites than it did at interimplantation sites. The luciferase report assay demonstrated that MMP26 is a target gene of miR-125b. And the expression profile of MMP26 showed an inverse relationship with miR-125b *in vivo* and *in vitro*. Overexpression of miR-125b in human EECs inhibited cell migration and invasion. Gain-of-function of miR-125b induced a significant decrease in the number of implantation sites. In conclusion, these data shed new light on how miR-125b triggers ER decline through the regulation of MMP26 function.

A receptive endometrium is one of the core factors for successful embryo implantation. The endometrium only exhibits a short period of receptivity (cycle days 20–24), known as the window of implantation (WOI)^1^,^2^. Attainment of endometrial receptivity (ER) requires the expression of a large number of cell-type-specific molecules and their potential functions^3^,^4^. Unfortunately, our knowledge of ER is limited, and there is a lack of biomarkers and methods to recognize or regulate the WOI. Understanding the molecular mechanisms of ER will help to improve the diagnosis and treatment of infertility.

The pregnancy rate following *in vitro* fertilization and embryo transfer (IVF-ET) has been improving steadily during the last 30 years, but remains unsatisfactory. It has been shown that elevated progesterone on the day of HCG administration is associated with reduced pregnancy rates^5^,^6^,^7^. The incidence of progesterone rise varies from 8% to 40% owing to the use of different test methods and cut-off levels^8^. However, elevated progesterone did not have a detrimental effect on oocyte quality in terms of fertilization and cleavage rates^9^. Embryo cryopreservation could be an approach to overcome the detrimental effect of elevated progesterone^10^. All these results indicate that elevated progesterone on the day of human chorionic gonadotrophin (HCG) administration may impair ER. The gene expression profile of the endometrium is significantly altered when the serum progesterone concentration is over 1.5 ng/ml^11^,^12^.

MicroRNAs are small non-coding RNAs (18–25nt) that regulate gene expression inversely by binding to the 3′ untranslated region (UTR) of target mRNAs at the posttranscriptional level; their effects include mRNA cleavage, translational repression or both^13^,^14^. MicroRNAs are involved in numerous biological processes, ranging from cell proliferation, cell differentiation, cell apoptosis to tumorigenesis^15^,^16^,^17^. In recent years, accumulated studies have suggested that microRNAs and their target genes play essential roles in embryo implantation and development^18^. Examples include miR-451/Ankrd46^19^, miR-429/Pcdh820^20^, miR-181/LIF^21^, miR-145/IGF1R^22^, miR-143/Lifr^23^, and let-7/mucin1^24^. MiR-125b is the human orthologue of lin-4, which is essential for embryonic proliferation and differentiation^25^. MiR-125b is expressed at high levels in the ovaries, pituitary gland, uterus and placenta, and has gained special interest in cancer research^26^. Embryo implantation shares various physiological and pathological events with cancer, however, its role in the establishment of ER is still unknown, especially for women with elevated progesterone on the day of HCG administration.

In this study, we clarified the expression of miR-125b in women with elevated progesterone on the day of HCG administration during embryo implantation, and demonstrated that MMP26, a member of the matrix metalloproteinase family, is the target gene of miR-125b. Gain-of-function of miR-125b also suppressed embryo implantation *in vivo*. The findings of our study may provide an experimental basis for better understanding of the molecular mechanisms involved in disturbance of ER.

## Results

### Expression of miR-125b in human endometrial epithelial and stromal cells

**Figure 1:**
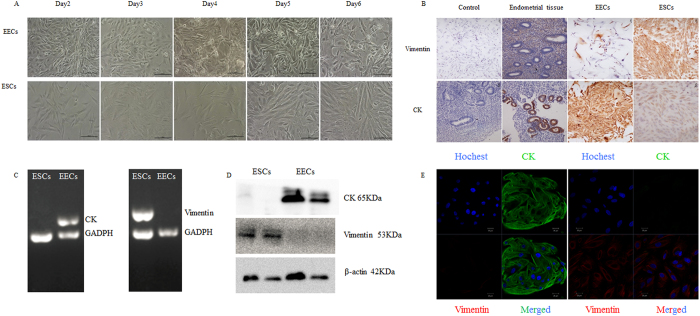
Identification of human endometrial epithelial cells and stromal cells. (**A**) Morphology of cultured endometrial cells for day 2–6. The upper were EECs and the under were ESCs. (**B**) Immunohistochemistry of endometrial tissue and endometrial cells. The first column were negative control without primary antibody in endometrial cells and endometrial tissue. The second column were endometrial tissue. The third column were EECs and the last column were ESCs. The top row showed dyeing with vimentin and the following row showed dyeing with cytokeratin (CK). (**C**) RT-PCR of Vimentin and CK in EECs and ESCs. (**D**) Western blotting showed EECs only expressed CK and ESCs only expressed vimentin. (The cropped gels were run under the same experimental conditions.) (**E**) Immunofluorescence of endometrial cells. The left were EECs and showed CK positive (green), the right were ESCs and showed vimentin positive (red), and nucleus were showed in blue. EECs: endometrial epithelial cells; ESCs: endometrial stromal cells.

Endometrial epithelial cells (EECs) and endometrial stromal cells (ESCs) may play a different role in ER. It would have obscured the cells’ specific functions if we had used the entire endometrial tissue as the sample. Hence, human EECs and ESCs were isolated separately and identified by morphology, immunohistochemistry, reverse-transcription PCR, western blotting and immunofluorescence (Fig. 1) using their specific markers, cytokeratin (CK) for EECs and vimentin for ESCs.

**Figure 2:**
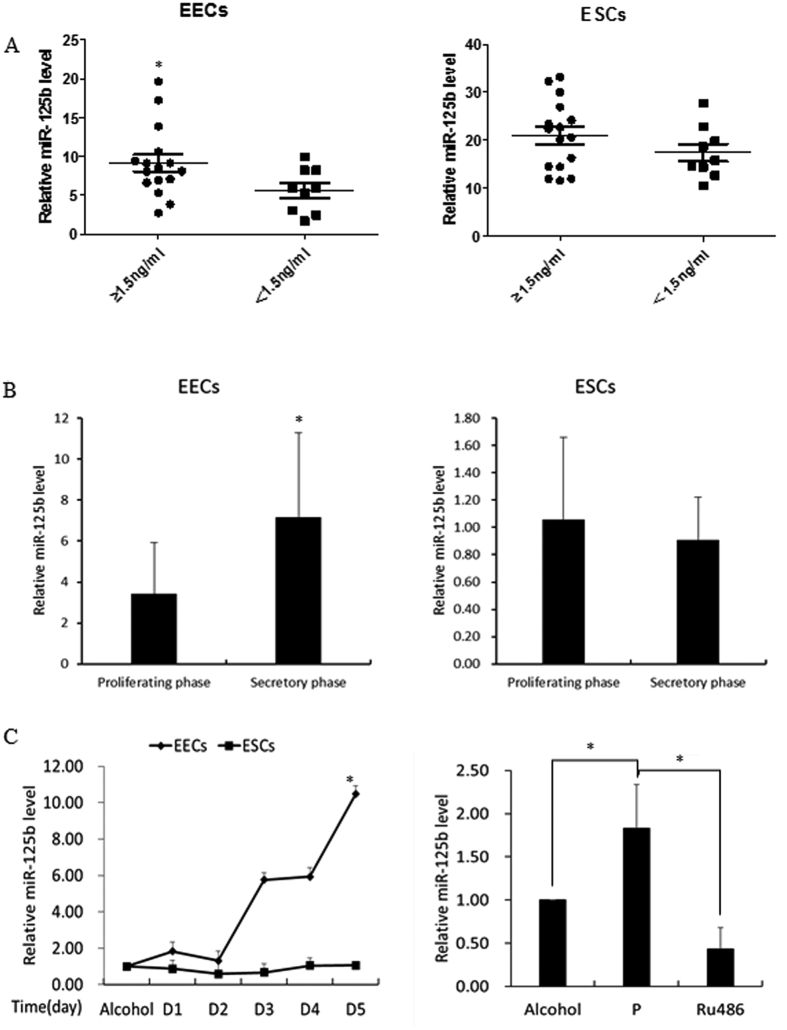
The expression of miR-125b in human endometrial epithelial cells and stromal cells. (**A**) The expression of miR-125b in elevated progesterone group (≥1.5 ng/ml) and normal progesterone group (<1.5 ng/ml) in EECs and ESCs. (**B**) The expression of miR-125b in different menstrual phase in EECs and ESCs. (**C**) The expression of miR-125b in *in vitro* cultured human EECs and ESCs when added 10–6 mol/L progesterone for five days. Ru486 could abrogate the increase of miR-125b in EECs. The value in alcohol group was arbitrarily set at 1 and data in other group were presented as ratios to the alcohol group. EECs: endometrial epithelial cells; ESCs: endometrial stromal cells. P: progesterone. Data were presented as mean ± SD, *p < 0.05.

On day of HCG+7, the expression of miR-125b in the elevated progesterone group (≥1.5 ng/mL evaluated on day of HCG+0) was significantly higher than in the normal progesterone group (<1.5 ng/mL evaluated on day of HCG+0) in EECs on day HCG+7, while no difference was observed in ESCs (Fig. 2A). To understand the expression profile of miR-125b in natural cycles, we examined the expression of miR-125b in different phases of menstruation. As shown in Fig. 2B, the level of miR-125b expression was much higher in the secretory phase than in the proliferative phase in EECs, while it was constant in ESCs. It is suggested that the expression of miR-125b in EECs may be influenced by periodic changes in estrogen and progesterone during the menstrual cycle.

In order to confirm this conjecture, we added 10^−6^ mol/L progesterone to *in vitro* cultured human endometrial cells, and the expression of miR-125b was indeed up-regulated in EECs and constant in ESCs. Furthermore, the progesterone antagonist Ru486 could abrogate the increase of miR-125b in EECs (Fig. 2C).

### Prediction and confirmation of miR-125b target genes

**Figure 3:**
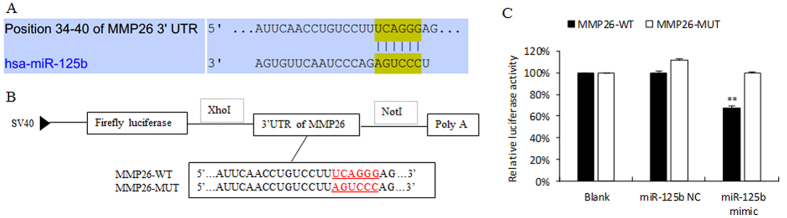
Prediction and confirmation of the miR-125b target gene. (**A**) The miR-125b binding site in the 3′UTR region of MMP26. (**B**) The 3′UTR fragment of MMP26 wild-type containing the binding site of miR-125b was cloned into the immediately downstream of firefly luciferase reporter gene. The putative binding site was mutated to the complementary nucleotides of MMP26 in the mutant-type. (**C**) Relative luciferase activity in 293T cells co-transfected with miR-125b mimic, miR-125b NC and MMP26-WT or MMP26-MUT vectors. The value in blank group was arbitrarily set at 100% and data in other group were presented as ratios to the blank group. Data were showed as mean±SD. **p < 0.01.

To predict the target gene of miR-125b, we used 10 online target prediction programs (DIANAmT, miRanda, miRDB, miRWalk, RNAhybrid, PICTAR4, PICTAR5, PITA, RNA22, Targetscan): MMP26 was one of the target genes predicted by five programs (Fig. 3A).

To verify the putative target gene, we performed the dual-luciferase reporter assay. The 3′UTR fragment of MMP26 containing the binding site was cloned immediately downstream of the firefly luciferase reporter gene in the psiCHECK^TM^-2 vector (MMP26-WT). In the mutant-type, the binding site was mutated to the complementary nucleotides of MMP26 (MMP26-MUT, Fig. 3B). MMP26-WT or MMP26-MUT reporter vectors were co-transfected with miR-125b mimics or miR-125b NC in 293 T cells. The luciferase activity in the group of co-transfection with miR-125b mimics and MMP26-WT led to a significant 32.32% decrease (p < 0.01) compared with the blank group. However, there was no obvious alteration in the group co-transfected with miR-125b mimics with MMP26-MUT (Fig. 3C). These results suggest that miR-125b may regulate gene expression by binding to the seed sequence in the 3′UTR region of MMP26.

**Figure 4:**
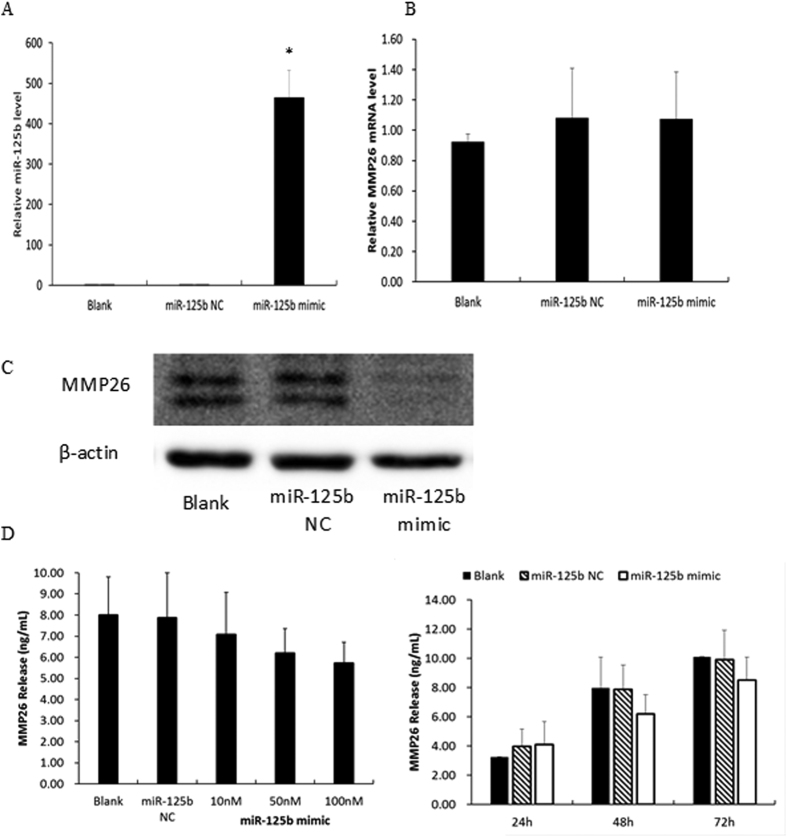
Confirmation of the target gene of miR-125b. (**A**) The expression of miR-125b after transfected human EECs with 50 nM miR-125b mimic or NC; (**B**) Expression of MMP26 mRNA in human EECs transfected with 50 nM miR-125b mimic or NC; (**C**) Expression of MMP26 protein in human EECs transfected with 50 nM miR-125b mimic or NC (The cropped gels were run under the same experimental conditions.); (**D**) Release of MMP26 in cell- culture medium after transfected with different concentration of miR-125b mimic and for different time. Data were presented as mean ± SD, *p < 0.05.

To further investigate whether MMP26 is the target gene of miR-125b, the expression of MMP26 mRNA and protein was examined in human EECs after transfection with miR-125b mimics, which could up-regulate miR-125b about 500-fold (Fig. 4A). Although MMP26 mRNA showed no change (Fig. 4B), the expression of MMP26 protein detected by western blotting showed a significant decrease (Fig. 4C). Similarly, the secretion of MMP26 in cell-culture medium, examined by ELISA, also showed an inverse correlation with miR-125b in a concentration- and time-dependent manner (Fig. 4D). These data suggest that miR-125b cannot degrade MMP26 mRNA, but it can inhibit MMP26 protein translation.

### The effect of miR-125b on cell migration and invasion

**Figure 5:**
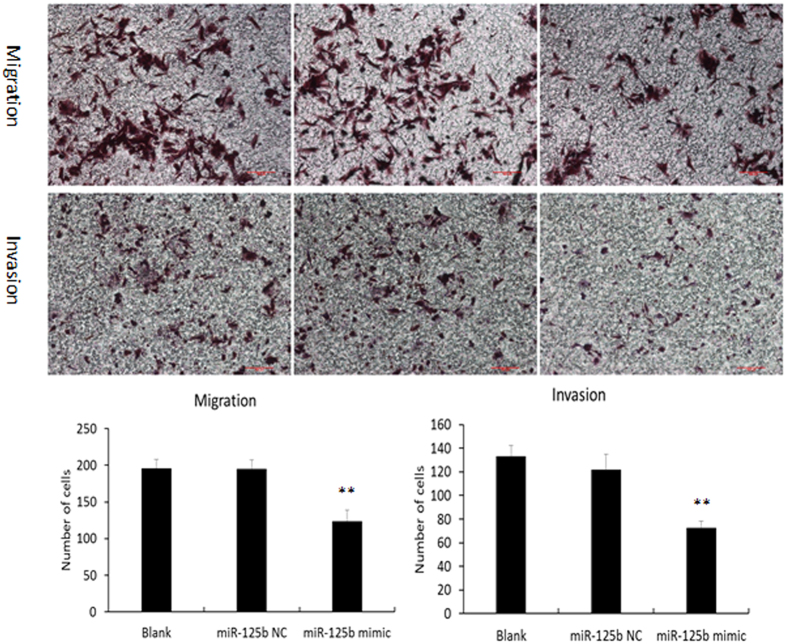
*In vitro* effect of miR-125b on migration and invasion capacity of human EECs by transwell assay. Data were presented as mean ± SD, **p < 0.01.

The effect of miR-125b on cell migration and invasion of human EECs was examined by transwell assay. As shown in Fig. 5, miR-125b mimics could significantly decrease the migratory and invasive capacity of human EECs. These results suggest that reduced secretion of MMP26 by miR-125b transfected human EECs may weaken cell movement ability.

### *In vivo* effect of miR-125b gain-of-function on embryo implantation

**Figure 6:**
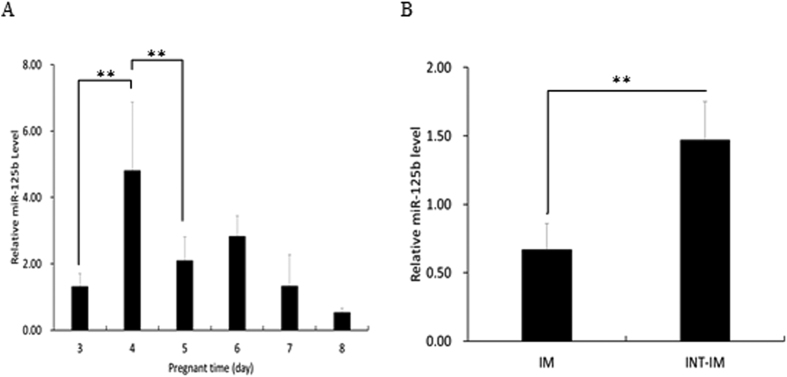
Expression pattern of miR-125b in mouse endometrial epithelial cells. (**A**) The expression profile of miR-125b during early pregnant time. It was highest on day 4, then decreased on day 5–8. (**B**) MiR-125b expression at implantation sites and interimplantation sites on day 5. Data were showed as mean ± SD. **p < 0.01. IM, implantation sites; INT-IM, interimplantation sites.

First, we examined the expression profile of miR-125b in EECs in untreated C57BL6/J mice during early pregnancy. Real-time PCR analysis showed that the level of expression of miR-125b was dramatically increased on day 4, the day before the implantation period (D5), and then decreased (Fig. 6A). Moreover, miR-125b expression showed a remarkable decrease at implantation sites when compared with interimplantation sites on day 5 (Fig. 6B). This suggests a close association of increased expression of miR-125b with the onset of embryo implantation, which occurs in the evening of day 4 in the mouse.

**Figure 7:**
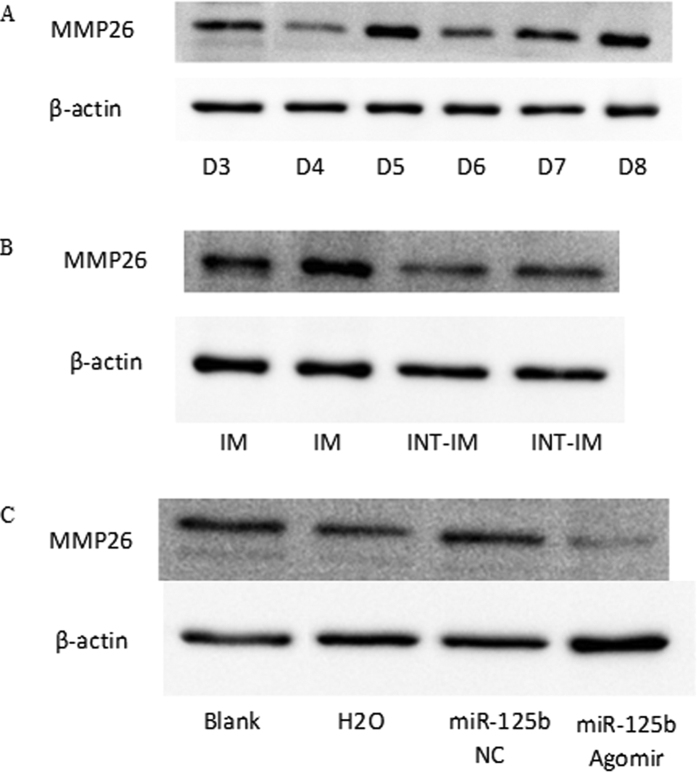
Expression of MMP26 protein in mouse EECs. (**A**) The expression of MMP26 protein in mouse EECs during day 3–8 of pregnancy; (**B**) The expression of MMP26 protein in mouse EECs at implantation sites and at interimplantation sites; (**C**) The expression of MMP26 protein in mouse EECs after cornu uteri injected with miR-125b agomir. EECs: endometrial epithelial cells; IM, implantation sites; INT-IM, interimplantation sites. (The cropped gels were run under the same experimental conditions).

In addition, the expression of MMP26 protein in mouse EECs showed an inverse correlation with miR-125b during days 3–8 (Fig. 7A), and was significantly higher at implantation sites than interimplantation sites (Fig. 7B). Furthermore, mouse uterine horns injected with miR-125b agomir showed decreased MMP26 protein *in vivo* (Fig. 7C).

**Figure 8:**
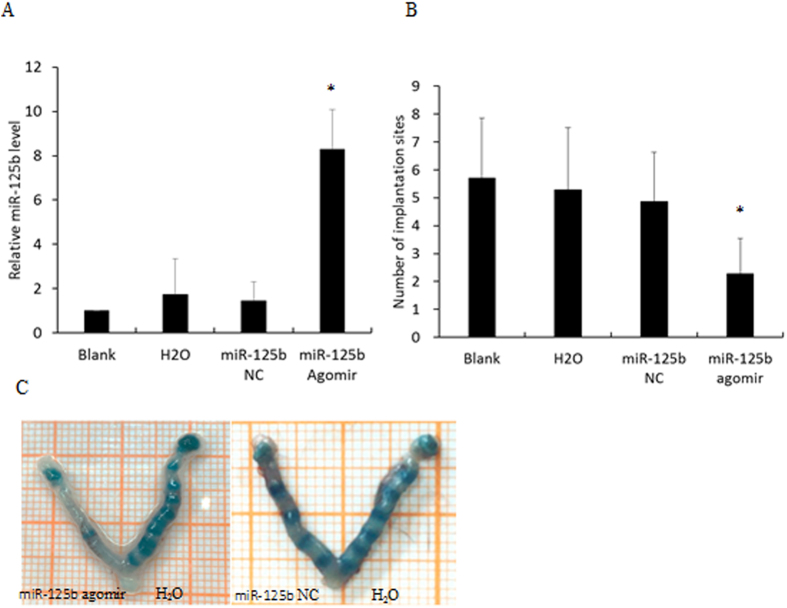
*In vivo* effect of miR-125b gain-of-function on embryo implantation. (**A**) The expression of miR-125b in mouse EECs after injecting miR-125b agomir, miR-125b NC and RNase-free water. (**B**) The number of implantation sites in each group. (**C**) The implantation sites were visualized as blue bands by caudal vein injection of 0.1 ml 1% Chicago Blue dye solution. Data were recorded as mean±SD, *p < 0.05.

To evaluate whether miR-125b can regulate embryo implantation, mouse uterine horns were injected with miR-125b agomir; these overexpressed miR-125b *in vivo*. The contralateral uterine horns were injected with RNase-free water on day 3 of pregnancy. In another group, miR-125b NC and blanks were treated, respectively, as controls. Real-time PCR showed that the injection of miR-125b agomir resulted in a significant increase of miR-125b expression (Fig. 8A). The implantation sites were identified by intravenous injection of Chicago Blue dye solution on day 5 or 7. The number of implantation sites in the miR-125b agomir group (2.29 ± 1.25) was significantly lower than those in the miR-125b NC group (4.86 ± 1.77; p < 0.05), water group (5.29 ± 2.22; p < 0.05) and blank group (5.71 ± 2.14), and there was no difference among the miR-125b NC group, water group and blank group (p > 0.05, Fig. 8B). It was demonstrated that up-regulation of miR-125b in EECs was detrimental to embryo implantation.

## Discussion

In the present study, our results found that miR-125b expression was up-regulated in the women with elevated progesterone on HCG administration day. Through *in-vitro* cell experiment and *in-vivo* mouse experiment, we found that miR-125b expression was depended on progesterone level and impaired embryo implantation, which gave an explanation for the decreased implantation rate in the women with elevated progesterone on HCG administration day. Furthermore we identified a miR-125b target gene, MMP26, and demonstrated that MMP26 is a key intermediary in the maintenance of ER regulated by miR-125b.

Progesterone can stimulate and regulate important cellular and tissue functions, playing a key role in maintaining pregnancy, preparing the body for conception and regulating the monthly menstrual cycle. In normal women, progesterone was gradually elevated since ovulation, and continuously elevated if fertilized embryo can implant into endometrium, but decreased if the implantation was failed. However, the implantation could be declined if the progesterone was elevated untimely. Evidence suggests that elevated progesterone on the day of HCG administration is associated with lower live birth rates^27^,^28^ and is more likely to happen in women with recurrent IVF failure^29^. From the perspective of ER, the gene expression pattern in the endometrium was significantly altered in women with serum progesterone over 1.5 ng/ml on the day of HCG administration during the WOI^11^,^12^.

Our study demonstrated that miR-125b was up-regulated in EECs from women with elevated progesterone compared to those with non-elevated progesterone. This result was different from that in the study of Li *et al.*^30^, perhaps because they used the whole endometrial tissue as the sample, while our study was specific at the cellular level. EECs and ESCs are the two main cell types in the endometrium, and our results showed that the expression pattern of miR-125b in the two cell types was very different. The expression of miR-125b in the secretory phase was significantly higher than in the proliferating phase in EECs and showed a progesterone-dependent manner *in vitro*. However, it was constant in ESCs. It has been suggested that elevated progesterone on the day of HCG administration up-regulates miR-125b in EECs and may be associated with a lower pregnancy rate.

MiR-125b is the human orthologue of lin-4, which is crucial for post-embryonic proliferation and differentiation^25^. Recently, miR-125b has gained special interest in cancer research^31^. On the one hand, miR-125b induces cell growth and proliferation, displaying oncogenic potential^32^,^33^,^34^,^35^. On the other hand, miR-125b is down-regulated in some tumor types, contributing to malignant transformation^36^,^37^,^38^. However, there has been no study of miR-125b in embryo implantation, which shares various physiological and pathological events with oncogenesis. MiR-125b is enriched in the ovaries, pituitary gland, uterus and placenta. Because of ethical considerations, we only examined the expression profile of miR-125b in mouse EECs during the peri-implantation period. The expression of miR-125b was significantly up-regulated in the preimplantation period (day 4), and down-regulated in the implantation period (day 5) and post-implantation period (days 7–8). In addition, miR-125b was expressed at lower levels at implantation sites than at interimplantation sites. The temporal and spatial expression patterns of miR-125b indicate its role in ER during the process of embryo implantation. Our study found that gain-of-function of miR-125b could reduce the number of implantation sites in mice.

MMP26 is a target gene of miR-125b, as predicted by bioinformatics programs and confirmed by the dual luciferase activity assay. MMP26 is a member of the matrix metalloproteinase family, which is involved in the degradation of extracellular matrix (ECM, such as type IV collagen, fibronectin, insulin-like growth factor-binding protein 1, etc.) in processes such as embryonic development, reproduction, and tissue remodeling. MMP26 is different from most MMP family members because it lacks a conserved C-terminal protein domain. MMP26 is an estrogen-sensitive gene and is co-expressed with estrogen receptor alpha in normal and pathological endometrium; an elevated progesterone level can exaggerate a decrease in MMP26 expression^39^,^40^. The expression of MMP26 in the endometrium in natural cycles is highest in the early secretory phase and decreased in the late secretory phase^41^. Our study found that the expression of MMP26 in EECs was 22.74-fold higher than in ESCs, but it could not be detected in the endometrial carcinoma cell line RL95–2 (data not shown). These findings are consistent with other studies that have shown that MMP26 expression is restricted mainly to normal EECs^42^,^43^. Therefore, we can only use human primary cultured EECs to verify the reverse relationship between miR-125b and MMP26 *in vitro*. MiR-125b could significantly down-regulate MMP26 protein both in cells and in secretions in culture medium, although it did not affect the expression of MMP26 mRNA. The results of the ELISA did not reach statistical significance, perhaps because of fewer replications and high individual variation, but it showed a decreasing trend. Additionally, the expression of MMP26 in mouse EECs showed an inverse relationship with miR-125b during days 3–8 of pregnancy.

The process of embryo implantation and placenta formation is associated with remodeling of the endometrium through the breakdown of ECM. MMP26 is essential for the degradation of ECM and participates in endometrium remodeling. Qiao *et al.* found that MMP26 was significantly decreased in patients with polycystic ovary syndrome (PCOS) during the WOI, indicating that this gene may affect ER in women with PCOS^44^. However, Altmae *et al.* pointed out that MMP26 was significantly up-regulated in women with unexplained infertility, when compared with fertile women^45^. Diaz-Gimeno *et al.* created a genomic diagnostic tool based on transcriptomic signature to evaluate ER. Among the 238 genes selected for the tool, MMP26 was included as a potential biomarker of ER^46^. Furthermore, Jiang *et al.* demonstrated that overexpression of MMP26 in an endometrial carcinoma cell line (Ishikawa cells) markedly promoted embryo attachment, and MMP26 was a novel downstream target gene of HOXA10^47^. In our study, we demonstrated that MMP26 is a target gene of miR-125b and that miR-125b could significantly down-regulate MMP26 *in vivo* and *in vitro*. Moreover, overexpression of miR-125b in human EECs can dramatically inhibit cell migration and invasion capacity by decreasing the expression of MMP26. This result was consistent with the reports of Xu *et al.*^48^, Li *et al.*^49^ and Wang *et al.*^50^ that miR-125b inhibited migration and invasion in cutaneous squamous cell carcinoma cells, non-small cell lung cancer cells and Ewing’s sarcoma cells. In addition, MMP26 plays an important role in invasion and metastasis of cancer cells^51^. Xu *et al.* found that MMP26 promoted cell invasion though the Wnt signaling pathway^52^. Zhang *et al.* demonstrated that silencing of MMP26 significantly retarded cell migration and invasion *in vitro*, and in part through coordination with MMP-9^53^. In endometrium, MMPs is necessary for embryo attachment and subsequent invasion, while suppression of MMPs is necessary for endometrial stability to prevent excessive invasion of extravillus trophoblasts^54^. Increased MMPs action has also been linked to implantation abnormalities and inflammatory milieu in endometrium^55^. Since progesterone regulation of endometrium during WOI involves a complex network and in this context the role of miR-125b and MMP26 in ER warrant further investigation.

In summary, our study demonstrated that miR-125b was significantly up-regulated in EECs from women with elevated progesterone during the WOI and showed a progesterone-dependent effect *in vitro*, whereas it was constant in ESCs. Similarly, the expression of miR-125b was significantly up-regulated in the preimplantation period, and down-regulated in the implantation period and post-implantation period, in mice. In addition, miR-125b showed a greater decrease at implantation sites than at interimplantation sites. MMP26 is a target gene of miR-125b. The expression profile of MMP26 showed an inverse relationship with miR-125b *in vivo* and *in vitro*. MiR-125b binds to the 3′UTR region of MMP26 and negatively regulates the expression of MMP26 by inhibiting mRNA translation, but does not affect mRNA stability. MiR-125b suppresses EEC migration and invasion by down-regulating MMP26, which may restrain embryo attachment and the subsequent invasion of the endometrium. Gain-of-function of miR-125b showed a significant decrease in the number of implantation sites. These findings give us a better understanding of the mechanisms involved in lower live birth rates in women with elevated progesterone on HCG priming day.

## Materials and Methods

### Patients and tissue collection

The study was approved by the Ethics Committee of Peking University Third Hospital and the methods were carried out in accordance with the approved guidelines. The written informed consent was obtained from all participants undergoing endometrial biopsies. Premenopausal women (aged 23–38 years) who asked for IVF-ET treatment due to male factor at the Reproductive Medicine Center, Peking University Third Hospital were recruited. All participants had a regular menstrual cycle and no positive findings in the uterus. Patients with other factors that may affect ER, such as polycystic ovary syndrome, endometriosis, ovarian tumor, hydrosalpinx and steroidal hormone treatment within the last three months, were excluded.

Endometrial samples were obtained from two groups of patients, with different experimental designs. First, controlled ovarian hyperstimulation was carried out using the GnRH agonist long protocol. Briefly, the participants were pretreated with GnRH agonist at the midluteal phase before the treatment cycle. Ovarian stimulation was started with human gonadotropin after confirmation of pituitary desensitization. Subsequently, HCG was administrated to trigger ovulation when the leading follicle reached a diameter of 18 mm. The patients were classified into two groups according to the serum progesterone level on HCG priming day (elevated P: ≥ 1.5 ng/mL; non-elevated P:<1.5 ng/mL). Endometrial biopsies were collected on day HCG+7. Women with elevated P had all embryos frozen in order to improve the implantation rate; women with non-elevated P did not have embryo transfer in a fresh cycle because of failure of fertilization. In this part, total 25 women were involved in, including 16 women with elevated progesterone, and 9 women with non-elevated progesterone on hCG administration day, respectively.

Second, we aimed to study the expression of miR-125b at different stages of menstruation during natural cycles. Endometrial samples were obtained in the proliferative phase or midluteal phase (LH+7), and divided into two parts: one was used for immunohistochemistry to confirm endometrial dating; the second was used for RNA extraction. In this part, total 32 women were involved in, including 16 endometrial samples in proliferative phase and 16 endometrial samples in secretory phase, respectively. LH was evaluated using urine.

### Isolation and culture of human endometrial epithelial and stromal cells

Human endometrial samples were transported to the laboratory in DMEM/F12 culture media (no phenol red, Invitrogen, Carlsbad, CA, USA) containing 100 U/mL penicillin and 100 μg/mL streptomycin (Invitrogen) on ice. After washing in Hanks buffered salt solution (HBSS, Invitrogen) three times, the endometrial tissues were carefully minced into 1 mm^3^ fragments and digested with 2 mg/ml collagenase type I (Life Technologies, New York, NY, USA) for 1 h, then treated with DNase I for 30 min, shaking every 10 min to achieve full digestion. The dissociated cellular suspension was filtered through a stacked sterile cell strainer assembly with 100 μm and 40 μm pore size (Corning, New York, USA) to separate EECs from ESCs. The EECs were retained in a 40 μm strainer, while the ESCs passed through the two strainers. The EECs were resuspended in DMEM/F12 supplemented with 10% fetal bovine serum (Charcoal/Dextran Stripped, Gemini, California, USA), and plated on Matrigel (BD, New Jersey, USA) coated 35 mm dishes (Corning). The ESCs were resuspended in culture media after removing red blood cells using ACK lysis buffer (Life Technologies, New York, NY, USA) and incubated at 37 °C in 5% CO_2_. Any EECs remaining with the ESCs were removed by selective adherence to culture dishes for 2 hours. Immunohistochemistry, reverse-transcription PCR, western blotting and immunofluorescence staining were performed to identify the purity of the isolated EECs and ESCs, using antibodies against cytokeratin 19 (ab52625, abcam, Cambridge, UK) and vimentin (ab8978, abcam, Cambridge, UK).

### Animal experiments

The animal experiments were approved by Animal Care and Treatment Committee of Peking University Health Science Center. Eight- to ten-week-old C57BL6/J mice (18–20 g) were provided by the Laboratory Animal Center of Peking University Health Science Center and housed in a specific-pathogen-free room with a 14:10 h light/dark controlled environment. The mice were mated with fertile males of the same strain at a ratio of 2:1, and the morning they presented a vaginal plug was considered as day one of pregnancy. Embryo implantation was assumed to occur on day 5. Mice were killed at 4 o’clock in the afternoon of days 3–8 (three per day) and EECs were isolated to extract protein and RNA. The implantation sites were identified by intravenous injection of 0.1 ml 1% Chicago Blue dye solution (Sigma-Aldrich, St. Louis, MS). On day 5, the mice were killed and the implantation and interimplantation sites were dissected carefully. The EECs were isolated for subsequent experiments.

To examine the influence of miR-125b on embryo implantation, naturally pregnant mice were randomly assigned to two groups (seven per group). MiR-125b agomir or negative control (NC) (RiboBio, Guangzhou, China) was injected into the right uterine horn, and RNase-free water was injected into the left uterine horn on day 3 at nine o’clock in the morning. On day 5 or 7, the mice were killed and the number of implantation sites was recorded.

### Immunohistochemical detection of cytokeratin and vimentin

Immunohistochemical staining was performed following previous study^56^. The biopsied endometrium samples were fixed in formalin and subsequently paraffin embedded. The sections were then incubated with primary antibody at 4 °C overnight. After that, the sections were treated with secondary antibody for 30 min. Diaminobenzidine was used as a chromogen, and the sections were counterstained with hematoxylin. The primary antibody was omitted to create negative controls. The primary antibodies were cytokeratin 19 (ab52625, abcam, Cambridge, UK) and vimentin (ab8978, abcam, Cambridge, UK).

### Immunofluorescence detection of cytokeratin and vimentin

EECs and ESCs were immunostained to detect the biomarker expression (cytokeratin and vimentin proteins), and scanning confocal microscopy^57^. Briefly, the cells were fixed in 4% paraformaldehyde for 30 minutes at room temperature, permeabilized with 1% Triton X-100 at 4 °C overnight, and blocked in 1% bovine serum albumin in phosphate-buffered saline (PBS) for 1 hour at room temperature. The cells were incubated with primary antibody in PBS at 37 °C with 5% CO_2_ in air for 1 hour. After washing three times in 0.1% Triton X-100 in PBS, cells were incubated with secondary antibody conjugated to flourescein isothiocyanate. for 1 hour at room temperature. The nuclear status of the cells was evaluated by staining with 10 m g/mL of propidium iodide for 10 minutes. The cells were then mounted on glass slides and examined with confocal laser-scanning microscopy (Zeiss LSM 710, Jena, Germany). The primary antibodies were cytokeratin 19 (ab52625, abcam, Cambridge, UK) and vimentin (ab8978, abcam, Cambridge, UK).

### Plasmid construction and dual-luciferase activity assay

MiRNAs regulate gene expression by binding to the 3′UTR of target mRNAs. The full length 3′UTR of MMP26 was amplified by PCR from human genomic DNA. The primers are listed as follows: forward/XhoI: 5′GGCGGctcgagCATACCTTAATGTTAGCAC3′; reverse/NotI: 5′AATgcggccgcGAGGACAAAATATTACTCT3′. The putative binding site was mutated to the complementary nucleotides of MMP26 in the mutant-type (wild-type: 5′UCAGGG3′; mutant-type: 5′AGUCCC3′). The amplified 3′UTR product was cloned immediately downstream of the firefly luciferase gene in the psiCHECK^TM^-2 vector (Promega, Madison, WI). The construction was verified by DNA sequencing.

For dual-luciferase reporter assays, 293T cells were seeded in 24-well plates, and transfected with 50 nM miR-125b mimics or NC (RiboBio), using Lipofectamine 2000 (Invitrogen), together with MMP26 wild-type or mutant-type 3′UTR reporter vector, respectively. After 48 h, the cells were harvested and the luciferase activity was measured with a dual-luciferase Reporter Assay System (Promega) according to the manufacturer’s instructions. The vector containing Renilla luciferase was co-transfected in each well for data normalization. Each treatment was performed in triplicate and the results were shown as the relative luciferase activity.

### Total RNA isolation and reverse transcription PCR

Total RNAs from endometrial biopsies and cells were extracted using TRIzol method following previous study^57^. For reverse transcription PCR, RNA samples were kept on ice, and then added the mixture that was consist of nonenzymatic components, reverse transcriptase, RNase inhibitor and DEPC water. The mixture was then put into a PCR machine. The program is that 10 min at 25 °C for primer incubation, 30 min at 48 °C for reverse transcription, and 5 min at 95 °C for reverse transcriptase inactivation. The resulting cDNA was diluted with TE-buffer and store at −20 °C. The cDNA was identified using agarose gel electrophoresis method.

### microRNA isolation and real-time PCR

microRNAs were enriched separately from Larger RNAs (>200nt) using the miRNeasy Micro Kit (Qiagen, Valencia, CA) according to the manufacturer’s instructions. The concentration and integrity of RNA were assessed using a NanoDrop 2000 spectrophotometer (thermo fisher scientific, New York, NY, USA). The miRNA was reverse transcribed into complementary DNA (cDNA) using a Thermo Scientific RevertAid First Strand cDNA Synthesis Kit with a specific RT primer (RiboBio, Guangzhou, China). The PCR was carried out in an ABI 7500 Sequence Detection System (ABI, Life Technologies, New York, USA) using SYBR Green Master Mix (Life Technologies, New York, NY, USA), and the relative level of expression was normalized to U6 or β-actin. Each sample was analyzed in triplicate. Data analysis was performed using the 2^−ΔΔCT^ method. The primer was shown in supplementary Table 1.

### Western blotting

Cells were lysed in RIPA lysis buffer and quantified with a method based on bicinchoninic acid. After boiling for 5 min, 40 μg protein from each sample was loaded on a 12% separating gel and 5% stacking gel and run at a constant 80 V for 30 min, followed by 120 V for 1.5 h. The protein was transferred to a polyvinylidene fluoride (PVDF) membrane at a constant 0.3 A for 70 min. The PVDF membrane was incubated with primary antibody overnight at 4 °C after blocking in 5% non-fat milk for 1 h at room temperature, then washed in TBST three times. The PVDF membrane was incubated with HRP-conjugated secondary antibodies at room temperature for 1 h. The protein bands were visualized using a SuperSignal® West Pico Trial Kit (Thermo); β-actin was used for normalization. The primary antibodies were cytokeratin 19 (ab52625, abcam, Cambridge, UK), vimentin (ab8978, abcam, Cambridge, UK), and MMP26 (ab200115, abcam, Cambridge, UK).

### Enzyme-linked immunosorbent assay (ELISA)

MMP26 is a secretary protein. In order to detect the secretion of MMP26 in cell culture supernatants, we used the MMP26 ELISA kit (Uscn Life Science, Wuhan, China), which can detect MMP26 at a range of 0.156–10 ng/mL. Before adding to the well, the supernatants were diluted 1:1 with Standard Diluent, according to the manufacturer’s protocol. Each group was analyzed in triplicate and assayed in three duplications.

### Migration and invasion assays

Human EECs were cultured in 24-well plates and transfected with miR-125b mimics or NC according to the manufacturer’s instructions (RiboBio). After 48 h, the cells were digested with trypsase and resuspended in serum-free medium. Migration and invasion assays were performed in a 24-well chamber (Corning). For invasion assays, before 5 × 10^5^/ml cells were seeded on the upper chamber, matrigel (BD) diluted 1:8 with serum-free culture medium was added to a membrane with 8 μm pores and incubated at 37 °C for 1 h. For the migration assays, 1 × 10^5^/ml cells were seeded on the upper chamber without matrigel. For both assays, 600 μl complete medium was added to the lower chamber. After incubation for 24 h, cells that passed through the membrane were fixed and stained with crystal violet (Sigma-Aldrich). The number of cells that crossed the membrane was counted in five random fields at 100x. Each experiment was performed in triplicate.

### Statistical analysis

Data recording and statistical analysis were performed with SPSS 19.0 (SPSS Inc, Chicago, Illinois). All continuous variables were presented as the mean ± SD and analyzed by independent-samples t test or one-way ANOVA. Statistical significance was considered positive if the P-value was less than 0.05.

## Additional Information

**How to cite this article**: Chen, C. *et al.* MiR-125b regulates endometrial receptivity by targeting MMP26 in women undergoing IVF-ET with elevated progesterone on HCG priming day. *Sci. Rep.* **6**, 25302; doi: 10.1038/srep25302 (2016).

## Supplementary Material

Supplementary Information
