# Supplementary material for: MiR-125b regulates endometrial receptivity by targeting MMP26 in women undergoing IVF-ET with elevated progesterone on HCG priming day
Source: Sci Rep. 2016 May 4;6:25302. doi: 10.1038/srep25302 (PMC4855158; doi:10.1038/srep25302)
Supplement: Supplementary Information [file srep25302-s1.pdf]

MiR-125b regulates endometrial receptivity by targeting MMP26 in women undergoing IVF-ET with elevated progesterone on HCG priming day

Cheng Chen[1,2], Yue Zhao[1,2,3], Yang Yu[1,2,3,*], Rong Li[1,2,3,*], Jie Qiao[1,2]

1 Reproductive Medical Centre, Department of Obstetrics and Gynecology, Peking University Third Hospital, Beijing, 100191, China

2 Key Laboratory of Assisted Reproduction, Ministry of Education, Beijing, 100191, China

3 Beijing Key Laboratory of Reproductive Endocrinology and Assisted Reproductive Technology, Beijing, 100191, China

* Corresponding author:

Supplementary table 1 Primer sequences for real time PCR.

Gene	Sequence 5'-3'
Cytokeratin	F: CTGACCGACGAGATCAACTTC
	R: TGGCGTTGGCATCCTTAAT
Vimentin	F: GATTCACTCCCTCTGGTTGATAC
	R: CTTGTAGGAGTGTCGGTTGTT
GADPH	F: GGCATTGTGGAAGGGCTCA
	R: GTGGATGCAGGGATGATGTTCT
MMP26	F: GGGACTTTGTTGAGGGCTATT
	R: GTCAAGTAGGTCTGTCCCATTC
β-actin	F: TGCCCATCTACGAGGGGTAT
	R: CTTAATGTCACGCACGATTTCC
